# Void Formation/Elimination and Viscoelastic Response of Polyphenylsilsesquioxane Monolith

**DOI:** 10.3390/ma11050846

**Published:** 2018-05-19

**Authors:** Yusuke Daiko, Yuki Oda, Sawao Honda, Yuji Iwamoto

**Affiliations:** Department of Life Science and Applied Chemistry, Nagoya Institute of Technology, Gokiso-cho, Showa-ku, Nagoya, Aichi 466-8555, Japan; daiko.yusuke@nitech.ac.jp (Y.D.); 28411042@stn.nitech.ac.jp (Y.O.); honda@nitech.ac.jp (S.H.)

**Keywords:** silsesquioxane, power module, void, viscoelasticity, breakdown voltage

## Abstract

Polyphenylsilsesquioxane (PhSiO_3/2_) particles as an organic-inorganic hybrid were prepared using sol-gel method, and monolithic samples were obtained via a warm-pressing. The reaction mechanism of particles’ polymerization and transformation to the monolith under the warm-press were investigated using solid state ^29^Si nuclear magnetic resonance (NMR) spectrometer, thermal gravimetric-differential thermal analyzer (TG-DTA), mass spectrometer (MS) and scanning electron microscope (SEM). Transparent and void-free monoliths are successfully obtained by warm-pressing above 180 °C. Both the terminal –OH groups on particles’ surface and warm-pressing are necessary for preparation of void-free PhSiO_3/2_ monolith. From the load-displacement measurement at various temperatures, a viscoelastic deformation is seen for PhSiO_3/2_ monolith with voids. On the other hand, an elastic deformation is seen for void-free PhSiO_3/2_ monolith, and the void-free monolith shows much higher breakdown voltage.

## 1. Introduction

An organic-inorganic hybrid of polyphenylsilsesquioxane (PPSQ, PhSiO_3/2_) has attracted much attention owing to its unique thermal softening and curing properties since Brown et al. reported the PhSiO_3/2_ as a ladder polymer with a stereoregular double chain structure in 1960 [1,2,3,4,5,6]. Various applications for a passivation layer for the InGaZnO transistor [6,7], hybridization with epoxy resin [8], beauty care [9], a rhodamine 6G-doped laser [10], framework of cage crystals [11,12] and gemini surfactant [13], have also been reported so far. PhSiO_3/2_ particle with monodispersed size was successfully prepared using a two-step acid-base catalyzed sol-gel process, and its size, molecular weight and glass transition temperature vary by controlling the sol-gel conditions such as the amount of added ethanol solvent and concentration/amount of acid-base catalysis [14,15]. One big advantage of PhSiO_3/2_ particle is that a monolithic bulk can be obtained by pressing the particles. For example, press-formed monolithic bulk electrolytes have been reported so far [16,17,18]. In the case for synthesis of a thick film with thickness >1 μm, crack generation is inevitable using conventional dip- or spin-coating sol-gel techniques, and thus repetitive dry/sintering and coating cycles are generally required. Kozuka succeeded in obtaining a crack free thick film without the repetitive coatings by utilizing a polyvinylpyrrolidone (PVP) assisted sol-gel method [19,20,21]. On the other hand, PhSiO_3/2_ particles possess a negative zeta potential (~ −30 mV) and those particles are deposited on conductive substrates via an electrophoretic deposition method, and thick films with arbitrary thickness and high transparency are obtained after a heat treatment [22,23].

We have investigated an application of PhSiO_3/2_ particles for an insulating layer of a power semiconductor. Ceramics with high thermal conductivity, such as AlN and Si_3_N_4_, have been used for the insulating layer [24,25]. Due to their susceptibility to crack generation during prolonged operation and their high fabrication cost, composite insulating layers consisting with epoxy resin and ceramic filler (e.g., boron nitride) for increasing thermal conductivity have also been developed so far [26]. From the strong demand of both the higher efficiency and energy saving of the power semiconductor, material shift from conventional silicon (Si) to silicon carbide (SiC) was gradually seen since the late 1980’s [27,28]. SiC-based power devices have various advantages including high frequency operation, high thermal conductivity, high breakdown voltage and high-temperature operating capability higher than 200 °C owing to its wide bandgap compared with silicon [29,30]. High temperature operation leads to decrease the volume of heat sink (cooling system). From the view point of increasing the operation temperature, we anticipated that PhSiO_3/2_ will be a candidate for the insulating layer instead of epoxy resin due to its high thermal stability.

For the insulating layer, void, which is one typical manufacturing-induced defect, adversely affects both the thermal conductivity and insulation property [31,32,33]. Also, voids can cause crack generation and crack growth [34,35]. Finite element computations for the correlation between voids and crack growth have been extensively studied [34,36]. Adriaensens et al. propose an nuclear magnetic resonance (NMR) imaging technique for detecting the number of voids in isobutylene-based elastomers [37]. The work presented here is aimed at in-situ monitoring void formation/elimination through the warm-pressing of PhSiO_3/2_ particles in order to prevent as possible the negative effects of voids. In this paper, we report the mechanism of PhSiO_3/2_ polymerization via warm-pressing and its relation with void formation/elimination. A hysteresis behavior between loading and unloading of the load-displacement measurement (viscoelastic to elastic transition) was found to be well correlated with the number of induced voids. We also found an enhancement of the alternating current (AC) breakdown voltage for the elastic-response samples.

## 2. Materials and Methods

Materials Phenyltriethoxysilane (PhTES) (98%) was purchased from Aldrich (St. Louis, MO, USA) and used as received without further purification. For hydrolysis reaction, PhTES (5 mL) was added drop by drop to ethanol (EtOH) and hydrochloric acid (0.01 mass %) mixture at room temperature, and the solution was stirred for 90 min. The solution was then added quickly to ammonia water (0.28 mass %) for reaction of dehydration condensation, and further stirred for 90 min. The molar ratio of PhTES:EtOH:H_2_O (in HCl solution):H_2_O (in NH_4_OH solution) was 1:10:40:120. The resultant PhSiO_3/2_ particles were washed by repeated dispersion in H_2_O and centrifugation and then dried at 60 °C [14,15,16]. Monolithic (pelletized) bulk sample was prepared by pressing the PhSiO_3/2_ particles at 120 MPa for 15 min using a metal mold (10 mm in diameter) at 25 °C, followed by increasing temperature to 120~280 °C and further pressed at 120 MPa for 15 min.

The morphology of samples was observed using a scanning electron microscope (JEOL, JSM-6010LA, Tokyo, Japan). A thermal gravimetric-differential thermal analyzer (TG-DTA) (Rigaku, TG8120, Tokyo, Japan) was used to measure the weight loss and glass transition temperature at a heating rate of 10 °C/min. The mass of gases from PhSiO_3/2_ was also studied by TG/DTA (Hitachi High Technologies Ltd., STA7200, Tokyo, Japan) and simultaneous mass spectrometry (JEOL, JMS-Q1500 GC, Tokyo, Japan) under flowing helium (100 mL/min) as a carrier gas with a heating rate of 10 °C/min. Solid state ^29^Si magic-angle spinning nuclear magnetic resonance (MAS NMR, Varian, Inc., Palo Alto, CA, USA) measurement was carried out using a Varian Unity 400 plus spectrometer. The NMR spectra were measured at 79.45 MHz, 6 μs of 90° pulse length, 30 s of decay time between pulses, and MAS spinning rate of 3.5 kHz.

**Figure 1:**
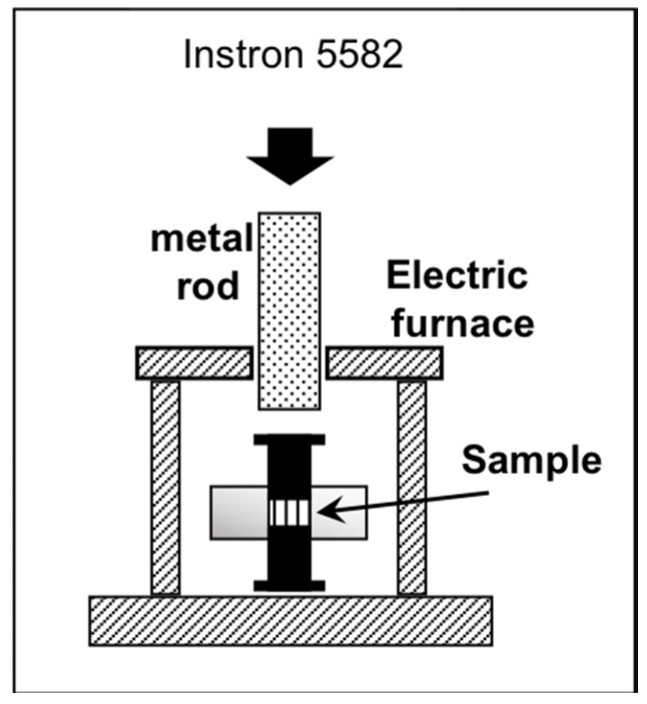
Schematic diagram of the setup for temperature-controlled load-displacement measurement using an Instron.

The load-displacement curves under both loading and unloading was measured using a testing machine (Instron 5582, Instron Japan company Ltd., Kanagawa, Japan). PhSiO_3/2_ particles were preliminary pressed at 5 kN and 25 °C using the aforementioned metal mold (10 mm in diameter), and then load-displacement curves were measured at 25, 120, 140, 160 and 180 °C. The load was increased up to 8 kN with cross-head speed of 0.005 mm/s, and then decreased to 0 N with the same speed. The loading-unloading cycle was repeated twice. A small electric furnace was used to control the temperature as shown in Figure 1. The alternate current (AC) breakdown voltage for PhSiO_3/2_ monolith was measured at 60 Hz using a dielectric strength tester (Tsuruga Electric Corporation, Model 8504, Osaka, Japan) at 25 °C and the measurement was performed inside Fluorinert (FC-40, 3M Japan Ltd., Tokyo, Japan) liquid in order to prevent an electric discharge of air. Silver paste electrodes (Dotite D-362, Fujikura Kasei Co., Ltd., Tokyo, Japan) were used. Five samples were prepared for each measurement. The voltage was increased stepwise (0.1 kV/step and 3 s of the interval time for each step). The voltage at the current over 0.5 mA was detected as a breakdown voltage.

## 3. Results and discussion

### 3.1. Structure and Degree of PhSiO_3/2_ Polymerization via Warm-Pressing

**Figure 2:**
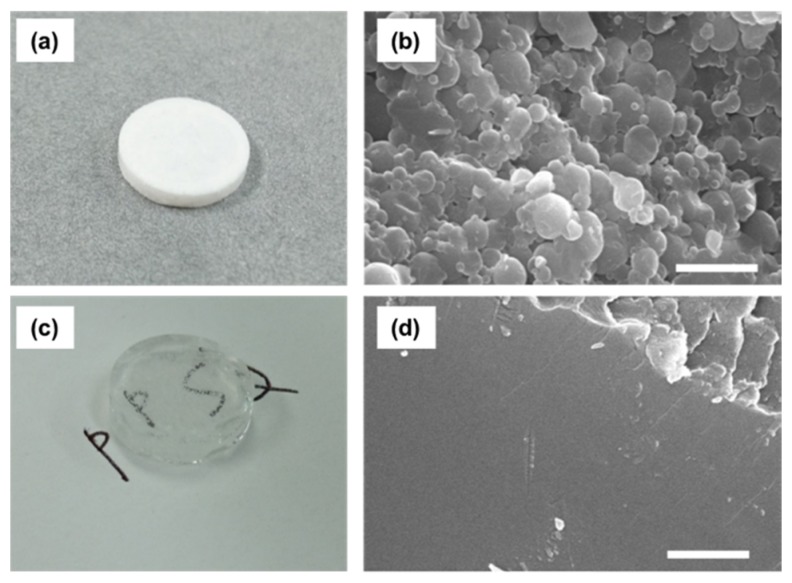
Photos and cross-sectional SEM images of monolithic samples prepared at 25 °C (**a**,**b**), and at 180 °C (**c**,**d**), respectively. Bars in SEM images indicate 5 μm.

The obtained PhSiO_3/2_ particles were approximately 1–2 μm in diameter. Monolith sample after pressing at 25 °C was opaque white, whereas that warm-pressing at 180 °C was transparent as shown in Figure 2a,c. However, many small bubbles were seen inside the monolith prepared at 180 °C. Cross sectional SEM images for both samples are also shown in Figure 2b,d. Although PhSiO_3/2_ particles are deformed under pressing at 25 °C, particles’ interfaces and voids between particles are clearly observed. On the other hand, such interfaces and voids decreased clearly for the monolith sample prepared at 180 °C.

**Figure 3:**
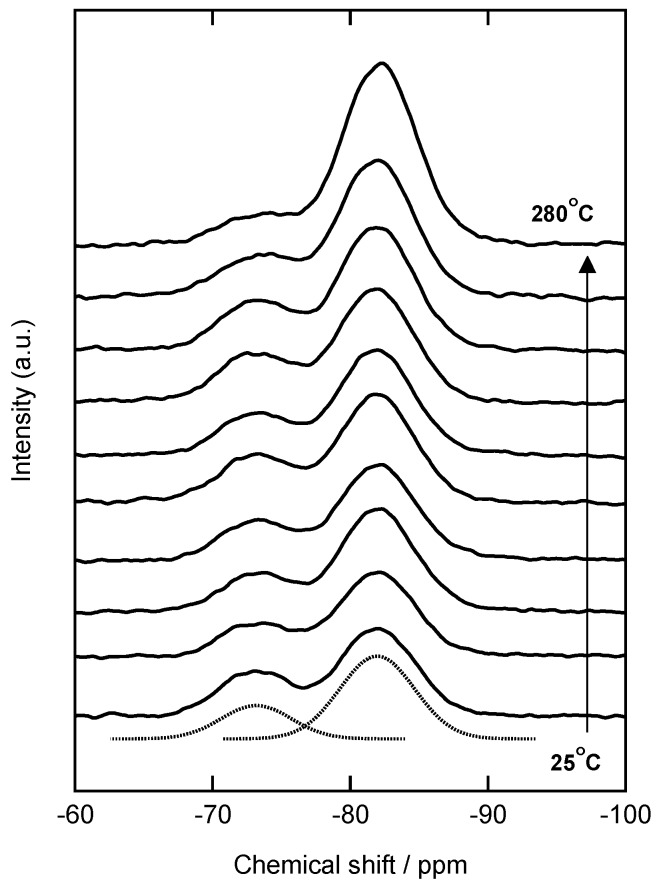
^29^Si NMR spectra for samples prepared via warm-pressing at 25, 140, 150, 160, 170, 180, 190, 200, 250 and 280 °C. Dotted lines at 25 °C are a result of peak deconvolution.

^29^Si NMR spectra for samples prepared at various temperature (warm-pressing) are shown in Figure 3. Two peaks are observed at −73 ppm and −82 ppm, which are assigned to T^2^ and T^3^ units, respectively (the *n* in T*^n^* indicates the number of bridging oxygen). In general, sol-gel-derived non-bridging oxygen in the T^2^ unit exists as terminal hydroxyl (–OH) or unreacted ethoxy (–OC_2_H_5_) groups. The peak intensity for the T^3^ unit increases with increasing the press temperature, suggesting the polymerization of PhSiO_3/2_ particles under the warm-pressing.

**Figure 4:**
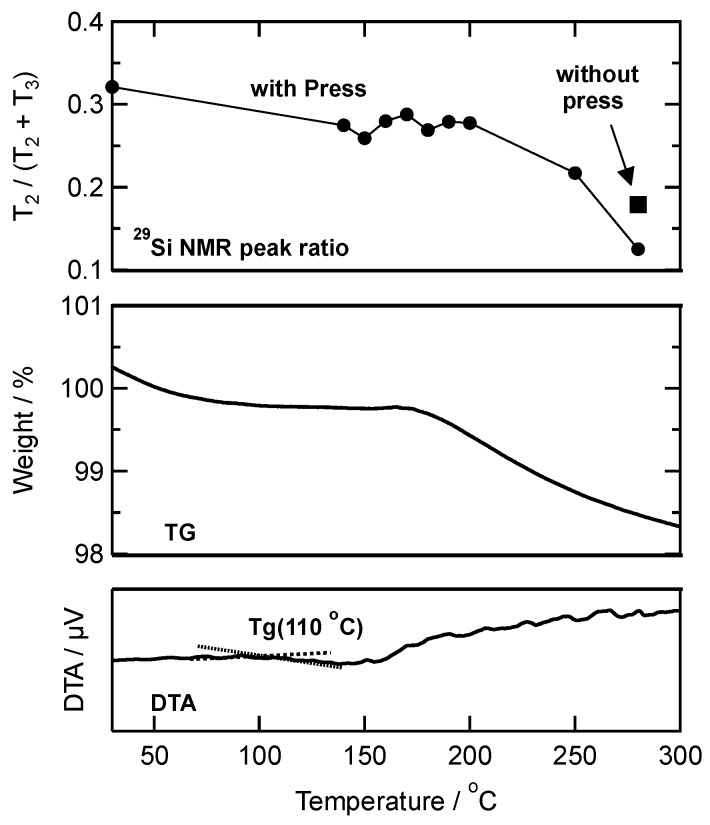
Changes in temperature of DTA and TG curves, and the ratio of T^2^ units calculated from ^29^Si NMR peak areas of T^2^ and T^3^ for PhSiO_3/2_ monoliths prepared with and without pressing.

**Figure 5:**
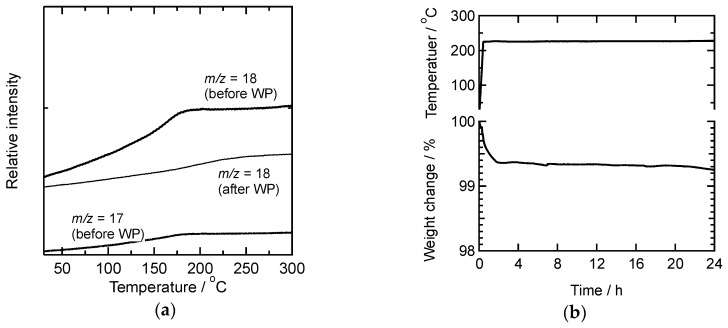
(**a**) Constituents of gaseous species for PhSiO_3/2_ particles as a function of temperature, and (**b**) changes in weight of a PhSiO_3/2_ monolith at 230 °C prepared by warm-pressing at 280 °C.

Takahashi et al. reported that the glass transition temperature (Tg) of PhSiO_3/2_ is in the range from 90 to 130 °C [15]. Figure 4 shows DTA curve of as-prepared PhSiO_3/2_ particles, and the Tg is estimated to be 110 °C. It is thus we adopted the temperature of warm-pressing above 110 °C. From the TG curve observed simultaneously with DTA, the weight loss of PhSiO_3/2_ particles up to 280 °C was calculated to be 1.3 wt %. The gas analysis was also carried out using a mass spectrometer, and results are shown in Figure 5a. Up to 280 °C, gaseous species, the *m/z* ratios of 18 and 17 were observed. These are assigned to H_2_O (a small relative intensity of the *m/z* = 17 is a fragment ion of H_2_O, which is also an evidence of water evaporation). No other molecules concerning with residual ethoxy groups such as OC_2_H_5_, C_2_H_5_ and CO_2_ were detected, suggesting the hydrolysis reaction of ethoxy groups proceeded completely and the aforementioned non-bridging oxygen exists as terminal hydroxyl groups.

Thermal stability was measured at 230 °C since SiC-based power devices can operate above 200 °C. As shown in Figure 5b, during a heat treatment at 230 °C for 24 h, a slight weight changes less than 0.1 wt % was confirmed for the monolith prepared by warm-pressing at 280 °C. The first weight loss from 100 to approximately 99.4 wt % under the heating is due to the dissociation of absorbed surface water.

The molar ratio of T^2^ and T^3^ units was estimated from a peak deconvolution of ^29^Si NMR peak area as shown dotted lines in Figure 3 (at 25 °C as an example), and calculated ratios of T^2^ for total silsesquioxane (T^2^ + T^3^) are also shown in Figure 4 together with DTA/TG results. Before the warm-pressing, the ratio of T^2^ was 0.32. The molecular weight for each monolith (*M*_monolith_) prepared at different temperatures was estimated as follows;

(1) $$f_{T2}=\frac{A_{T2}}{A_{T2}+A_{T3}},\;f_{T3}=\frac{A_{T3}}{A_{T2}+A_{T3}}$$

(2) $$M_{\mathrm{monolith}}=f_{T2}\times M_{T2}+f_{T3}\times M_{T3}$$

where *A*_T2_ and *A*_T3_ are the ^29^Si NMR peak areas, *f*_T2_ and *f*_T3_ are the molar fractions, and *M*_T2_ (138.2 g/mol) and *M*_T3_ (129.2 g/mol) are the molecular weights for T^2^ and T^3^ units, respectively. For example, for 1 mol of PhSiO_3/2_ particles, the sample weight for as-prepared PhSiO_3/2_ particle is 0.32 × 138.2 + 0.68 × 129.2 = 132.07 g, whereas the sample weight after heating at 280 °C (without pressing) is 0.18 × 138.2 + 0.72 × 129.2 = 130.81 g. Note that the change in the T^2^ fraction as a function of temperature is in good agreement with the weight loss curve (TG) (Figure 4). Weight loss is estimated to be approximately 1.0 wt % based on the result of ^29^Si NMR, which agree well with the result of TG (1.3 wt %). Also, the fraction of T^2^ decreases effectively by pressing (*f*_T2_ = 0.12) as compared with that prepared without pressing (*f*_T2_ = 0.18).

**Figure 6:**
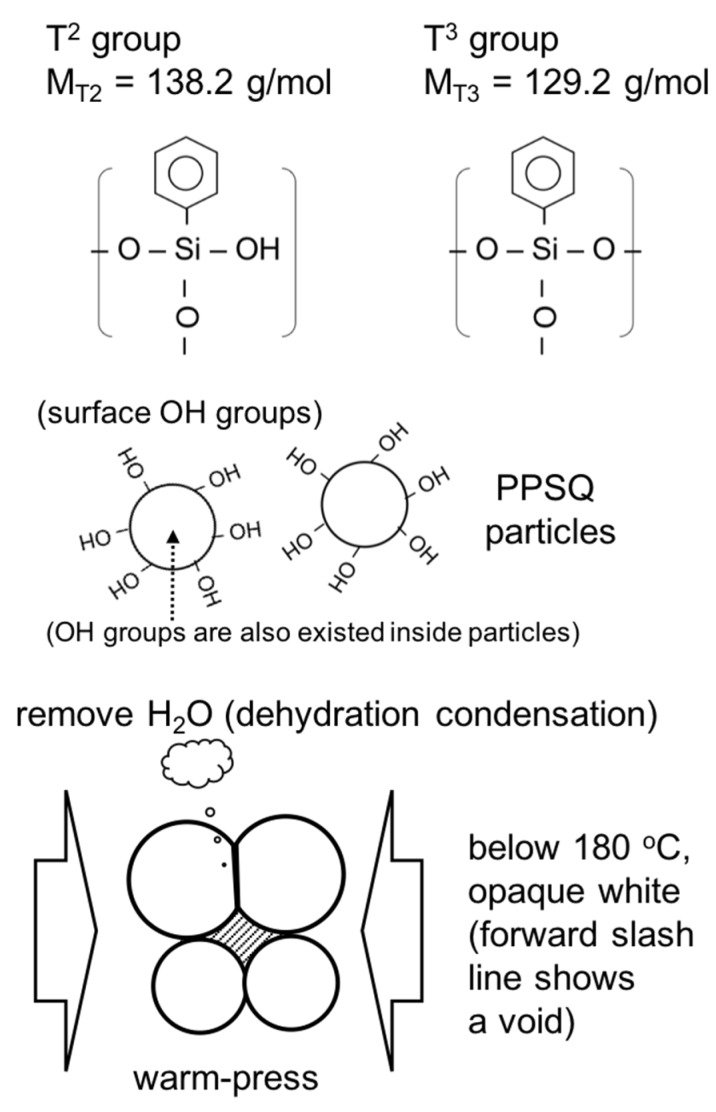
Schematic diagram of dehydration condensation of PhSiO_3/2_ particles.

From these results, the reaction of the monolith formation of PhSiO_3/2_ particles under the warm-pressing is progressed as shown in Figure 6. Hydroxyl groups as the non-bridging oxygen of T^2^ on particle surface react and the dehydration condensation occurs, resulting into the evaporation of H_2_O gas and which cause the formation of bubbles inside the monolith as shown in Figure 2c. These bubbles degrade the insulation property, and a slow increase for approximately a few min up to 120 MPa was effective in order to eliminate bubbles (to remove H_2_O gas). The dehydration condensation reaction promotes effectively by the warm-pressing owing to increase the contact area of PhSiO_3/2_ particles. After the warm-pressing, the amount of evaporated H_2_O decreases significantly. This is also an evidence of the dehydration condensation reaction during the warm-pressing. Both the terminal –OH groups on particles’ surface and warm-pressing above the glass transition temperature are necessary for preparation of void-free PhSiO_3/2_ monolith. Since we obtain a void-free PhSiO_3/2_ monolith at 180 °C, T^2^ units (–OH) remaining above 180 °C are considered to be mainly placed inside PhSiO_3/2_ particles which is not removed at temperatures up to 280 °C.

### 3.2. Loading-Unloading Hysteresis and Void Formation

**Figure 7:**
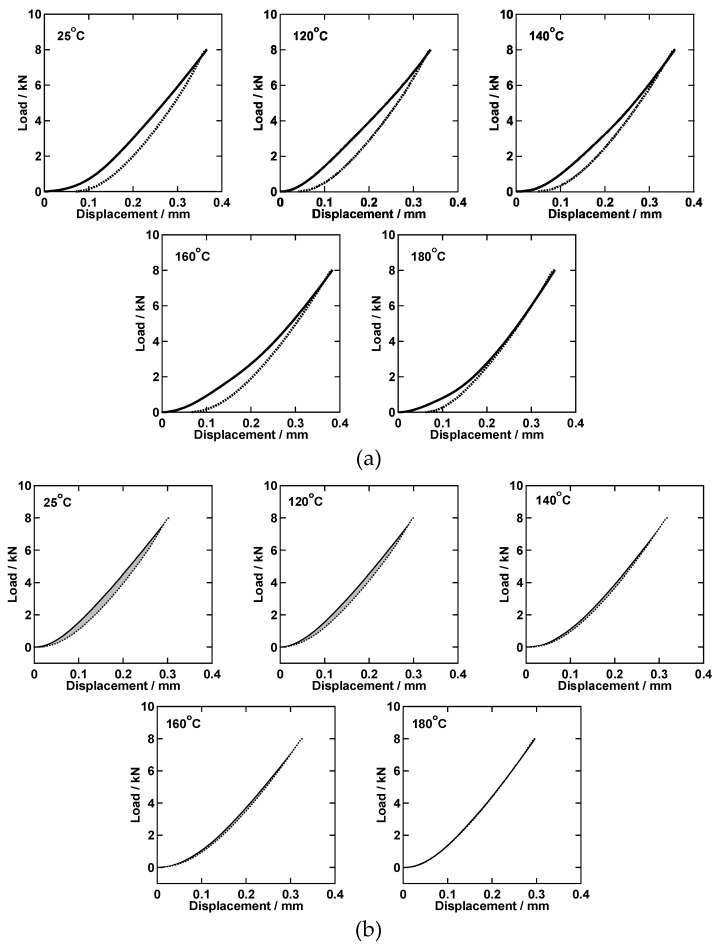
Load-displacement curves measured at various temperatures (**a**) the first loading-unloading cycles and (**b**) consecutive second loading-unloading cycles (solid line: loading, dotted line: unloading).

The deformation of PhSiO_3/2_ particles was confirmed by SEM observation, even at room temperature pressing. However, particles’ interfaces and voids are disappeared after warm-pressing above 180 °C. The mechanical response for the structural changes under the reaction of dehydration condensation was measured using an Instron. Figure 7a shows the load-displacement curves measured at temperatures ranging from 25 to 180 °C. The displacement for the metal mold and rod are not considered. In the case for the first loading-unloading cycle, a plastic deformation was observed for all temperatures, which is attributed to the deformation of PhSiO_3/2_ particles. The consecutive second loading-unloading cycles at the same temperature are shown in Figure 7b. The plastic deformation was not observed, and the displacement for unloading recovered to 0 mm at 0 kN. Note that a hysteresis between the loading and unloading is clearly seen except the measurement at 180 °C. This hysteresis is corresponding to a typical viscoelastic deformation. At 180 °C, the unloading curve was completely overlapped with the loading one, suggesting an elastic deformation.

Linear viscoelastic models are made up of linear springs and linear viscous dashpots [38]. The linear spring and dashpot elements are expressed as follows.

*σ* = *E·ε*  (spring) (3)

*σ* = *η·*d*ε*/d*t*  (dashpot) (4)

where *σ* is the stress, *ε* is the strain, *E* is the Young’s modulus and *η* is the coefficient of viscosity. At the first loading, a plastic deformation of PhSiO_3/2_ particles occurs and a monolith is obtained. Below 180 °C, samples are partially opaque white because of the unsatisfied dehydration condensation (void formation).

On the other hand, a transparent and void free monolith was obtained at 180 °C, and the second cycle behavior of which can be expressed only by a spring element. These results strongly suggest that the viscoelastic response induced by the dashpot element at the second cycle is correlated strongly with voids between PhSiO_3/2_ particles’ interfaces.

**Figure 8:**
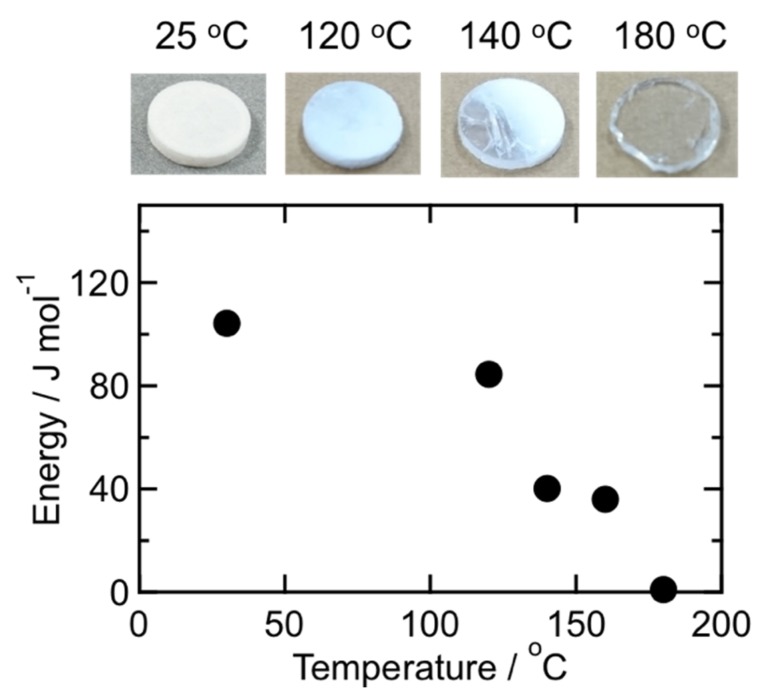
Relationship between the warm-pressing temperature and the deformation energy corresponding to the hysteresis area shown as grey color in Figure 7 (2nd cycle).

**Figure 9:**
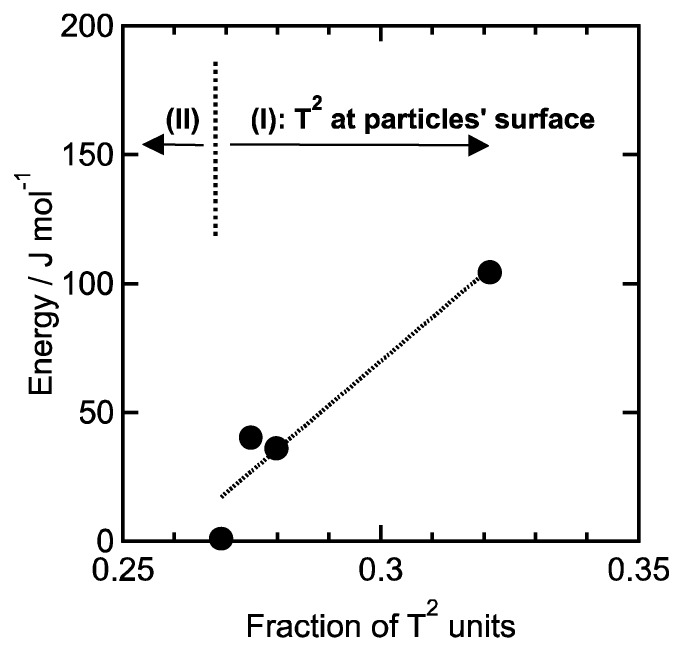
Relationship between the fraction of T^2^ units and the deformation energy. The fraction above 0.27 (region I) is corresponding to the –OH groups placed at particles’ surface, and below 0.27 (region II) is that placed inside particles.

The hysteresis area of the second loading-unloading cycle is corresponding to a deformation energy, and the energy for 1 mol of PhSiO_3/2_ was estimated from the hysteresis area by integration of loading and unloading curves. Figure 8 shows the relationship between the pressing temperature and the deformation energy. Note that the temperature dependence of the energy is also in good agreement with the fraction of T^2^ units as well as the weight loss shown in Figure 4. As above mentioned about the mechanism of hydration condensation, –OH groups exist on PhSiO_3/2_ particles’ surface, and such surface –OH groups remaining after the warm-pressing should be observed around voids. We assumed that the number of voids and remaining -OH groups is correlated directly with each other. As shown in Figure 9, a good linear correlation is successfully obtained between the deformation energy and the fraction of T^2^ unit (= –OH groups). The viscoelastic deformation energy estimated from the hysteresis of loading-unloading curves is an effective parameter in order to in-situ monitor the formation/elimination of voids during the warm-pressing.

Further investigations, including the time dependences and the stress relaxation under the warm-pressing, are in progress. Insulating resins for power modules, ceramic fillers are mixed together to increase the thermal conductivity, and voids are often induced at resin/filler interfaces. We have also investigated about the mechanical response for organosilsesquioxane and ceramic filler composites using the aforementioned technique, and a similar hysteresis behavior was confirmed. These results will be reported elsewhere.

### 3.3. AC Insulating Property

The breakdown voltage of the PhSiO_3/2_ monolith was measured at 25 °C in order to clarify the effect of voids on the voltage. PhSiO_3/2_ monoliths warm-pressed at 140 °C showed the breakdown voltage around 4.8 kV/mm. On the other hand, the voltage improves higher than 15 kV/mm for all monoliths warm-pressed at 180 °C. The maximum voltage of the dielectric strength tester is 10 kV, and we could not estimate the breakdown voltage with high accuracy for 180 °C samples. The breakdown voltage above 30 kV (0.5 mm thickness) was reported for epoxy resin/alumina composite insulators [39]. As discussed in Figure 8, voids are existing in samples prepared at 140 °C, whereas samples prepared at 180 °C are void free based on the results of viscoelastic measurement. These voids cause a partial discharge, leading to an electric breakdown [40]. Effective enhancement of the AC breakdown voltage for the elastic-response samples are experimentally confirmed. Mechanical responses using the similar technique for filler and olganosilsesquioxane composites and detailed analysis about the dashpot element will be reported soon.

## 4. Conclusions

Polyphenylsilsesquioxane (PhSiO_3/2_) monoliths were prepared using sol-gel-derived PhSiO_3/2_ particles by a warm-pressing, and the condensation reaction, void formation/elimination and insulating property were investigated. The monolith is formed through a dehydration condensation between –OH groups on particles’ surface. A deformation of PhSiO_3/2_ particles was seen even at 25 °C at 120 MPa of the pressure, however the obtained monolith was opaque white and particles’ interfaces and voids were clearly observed. A void free monolith was successfully obtained by the warm-pressing of PhSiO_3/2_ particles above 180 °C. The fraction of –OH groups (T^2^ units) and a deformation energy induced by a dashpot element were estimated from the ^29^Si NMR and the hysteresis area of loading-unloading curves, respectively. It was found that there is a good linear relationship between them. Compared with the observation of voids utilizing other techniques such as SEM or porosity analysis, this hysteresis analysis has a significant benefit since the results about void formation and elimination are obtained simultaneously during PhSiO_3/2_ particles’ warm-pressing, and which can be applied for various manufacturing-induced defects.
